# Dietary Observations of Ultra-Endurance Runners in Preparation for and During a Continuous 24-h Event

**DOI:** 10.3389/fphys.2021.765888

**Published:** 2021-11-24

**Authors:** Emma J. Kinrade, Stuart D. R. Galloway

**Affiliations:** ^1^Department of Occupational Therapy and Human Nutrition and Dietetics, School of Health and Life Sciences, Glasgow Caledonian University, Glasgow, United Kingdom; ^2^Physiology, Exercise and Nutrition Research Group, Faculty of Health Sciences and Sport, University of Stirling, Stirling, United Kingdom

**Keywords:** multiple transportable carbohydrates, sport, continuous glucose monitors, exercise, nutrition

## Abstract

Carbohydrate (CHO) intake recommendations for events lasting longer than 3h indicate that athletes should ingest up to 90g.h.^−1^ of multiple transportable carbohydrates (MTC). We examined the dietary intake of amateur (males: *n*=11, females: *n*=7) ultra-endurance runners (mean age and mass 41.5±5.1years and 75.8±11.7kg) prior to, and during a 24-h ultra-endurance event. Heart rate and interstitial glucose concentration (indwelling sensor) were also tracked throughout the event. Pre-race diet (each 24 over 48h) was recorded *via* weighed intake and included the pre-race meal (1–4h pre-race). In-race diet (24h event) was recorded continuously, in-field, by the research team. Analysis revealed that runners did not meet the majority of CHO intake recommendations. CHO intake over 24–48h pre-race was lower than recommended (4.0±1.4g·kg^−1^; 42±9% of total energy), although pre-race meal CHO intake was within recommended levels (1.5±0.7g·kg^−1^). In-race CHO intake was only in the 30–60g·h^−1^ range (mean intake 33±12g·h^−1^) with suboptimal amounts of multiple transportable CHO consumed. Exercise intensity was low to moderate (mean 68%HR_max_ 45%VO_2max_) meaning that there would still be an absolute requirement for CHO to perform optimally in this ultra-event. Indeed, strong to moderate positive correlations were observed between distance covered and both CHO and energy intake in each of the three diet periods studied. Independent *t*-tests showed significantly different distances achieved by runners consuming ≥5 vs. <5g·kg^−1^ CHO in pre-race diet [98.5±18.7miles (158.5±30.1km) vs. 78.0±13.5miles (125.5±21.7km), *p*=0.04] and ≥40 vs. <40g·h^−1^ CHO in-race [92.2±13.9miles (148.4±22.4km) vs. 74.7±13.5miles (120.2±21.7km), *p*=0.02]. Pre-race CHO intake was positively associated with ultra-running experience, but no association was found between ultra-running experience and race distance. No association was observed between mean interstitial glucose and dietary intake, or with race distance. Further research should explore approaches to meeting pre-race dietary CHO intake as well as investigating strategies to boost in-race intake of multiple transportable CHO sources. In 24-h ultra-runners, studies examining the performance enhancing benefits of getting closer to meeting pre-race and in-race carbohydrate recommendations are required.

## Introduction

Ultra-endurance running presents the athlete with a substantial nutritional challenge. With distances up to, and in excess, of 100miles and time limited events, such as 24-h races, it is vital that strategies are enforced to delay or minimise fatigue. Nutrition can play a key role in the preparation for, and execution of, ultra-endurance races at any level of competition. Current recommendations for ultra-endurance activities consider both pre-race diet and intake during events. Intakes of 8–12g·kg^−1^ CHO per 24-h are recommended in the 36–48h leading up to a prolonged endurance event to ensure well stocked muscle glycogen, with a further 1–4g·kg^−1^ in a pre-race meal during the final 1–4h recommended to top up liver glycogen stores (Thomas et al., 2016; Costa et al., 2019a). During prolonged exercise (>2h), exogenous CHO ingestion can prevent hypoglycaemia, maintain high rates of CHO oxidation, and increase endurance capacity (Jeukendrup, 2014). Recommendations for endurance activities lasting >2.5–3h, are to consume up to 90g·h^−1^ of multiple transportable carbohydrates (MTC; Burke et al., 2011). This amount can prove challenging in ultra-endurance running events (Costa et al., 2019a,b) although higher intakes of 120g·h^−1^ are possible and reduced exercise-induced muscle damage, in elite mountain-marathon runners (Viribay et al., 2020).

Studies investigating CHO intake of ultra-runners during competition have shown large variations in intake (25–71g·h.^−1^), at elite and non-elite levels (Glace et al., 2002; Moran et al., 2011; Stuempfle et al., 2011; Costa et al., 2014; Wardenaar et al., 2015; Stellingwerff, 2016; Martinez et al., 2018; Lavoué et al., 2020). Faster/elite runners have been shown to consume more hourly CHO than slower/amateur runners (Stellingwerff, 2016), and finishers reported to consume more than non-finishers (Stuempfle et al., 2011). From these studies, a higher CHO intake is associated with improved performance, but ultra-runners typically consume lower amounts than recommended, and less than competitors in other ultra-endurance disciplines (Pfeiffer et al., 2012). While these previous studies present evidence of actual CHO intake during ultra-endurance events, there is a lack of information on the mix of CHO sources ingested (i.e., amounts of glucose, sucrose, fructose, lactose, galactose, maltose, starch, or maltodextrin consumed), as well as frequently little indication of pre-race CHO intakes. The benefits of ingesting MTC include less gastrointestinal (GI) complaints at high CHO ingestion rates (Costa et al., 2017; Miall et al., 2018), and increased exogenous CHO oxidation rates (Wilson, 2015). The few studies examining MTC intake of runners have shown no convincing performance benefits, unless used as part of a gut-training protocol (Costa et al., 2017) although these have not specifically focused on ultra-endurance events (Pfeiffer et al., 2009; Lee et al., 2014).

Current recommendations for CHO intake rise with increasing exercise duration, but exercise intensity should also be considered. It is often reported that the rate of CHO intake should likely be reduced for those performing at lower intensities (Jeukendrup, 2014). However, for ultra-runners competing in events lasting greater than 10–12h, it would seem that carbohydrate ingestion rates should probably match those recommended for shorter events (~3–4h), to help meet the considerable metabolic demands of sustained activity. Although fat oxidation may provide much of the fuel utilised in events lasting up to 24h, there will be an absolute requirement for CHO to spare muscle and liver glycogen stores, and to maintain blood glucose concentration, in order to sustain intensity of activity over that duration. Maximising CHO availability before and during such events is therefore a key to maintaining performance (Williamson, 2016). Achieving desired CHO intake during an ultra-endurance run will require intake of MTC’s, and intake should be tailored to individual athletes’ tolerance levels (Stellingwerff and Cox, 2014) with higher rates of MTC intake being tolerable following appropriate gut-training (Costa et al., 2017). However, no studies have closely examined both pre-race CHO intake and in-race CHO sources ingested by ultra-endurance runners over a 24-h event.

The present study therefore investigated dietary intakes of ultra-endurance runners prior to and during a competitive 24-h event. We aimed to assess pre-race CHO intake, to describe the mix of individual CHOs consumed by participants’ in-race, and to evaluate the potential requirement for future MTC intervention strategies. We also aimed to assess in-race glycaemic responses in relation to feeding strategies. We hypothesised that amateur ultra-distance runners would fall short of recommended CHO intake targets before and during the event, and that, intake of MTC’s could be improved during the event.

## Materials and Methods

### Study Participants and Event Details

Eighteen amateur ultra-endurance runners (males: *n*=11, females: *n*=7) in the Glenmore-24 (G24) trail race (Aviemore, Scotland) agreed to participate in the study. G24 is a continuous undulating trail race on forest trails and tracks, over repeated laps of 4miles (6.4km), where the winner travels the furthest distance in 24-h. The event begins at 12 noon and ends after 24-h with runners able to change to a smaller 0.25miles (400m) grass field for the final hour of the event. Each large lap consists of approximately 80m (270feet) of ascent and descent.

Inclusion criteria for participants were: males or females; aged 18–50years; completed at least one previous ultra-marathon event. We specifically aimed to recruit a sample that was representative of the full range of competitors at the event. Participant characteristics are shown in Table 1. Ethics approval was granted by University of Stirling Ethics of Research Committee. All participants gave written informed consent prior to study commencement. Of the 18 participants, 15 (11 male, four female) performed an incremental maximal treadmill test at the University laboratories to enable VO₂_max_ and HR_max_ to be identified. The protocol used for the VO₂_max_ test involved participants starting at 8km/h (females) or 10km/h (males) on a 1% gradient, increasing speed in the first few stages before increasing gradient by 2% each minute until volitional fatigue.

**Table 1:** Participant anthropometric data, VO_2max_, ultra-endurance running experience and nutritional composition of the pre-race diet, pre-race meal prior to the start of a 24-h ultra-endurance race, and in-race data (distance covered, pace, mass loss, heart rate, and relative %HR_max_ and %VO_2max_) by *n*=18 participants during the Glenmore 24 trail race.

Variable	All participants (*n*=18)	Males (*n*=11)	Females (*n*=7)
Age (years)	41.5±5.1	39.3±4.1	45.0±4.7
Weight (kg)	75.8±11.7	81.7±6.6	66.5±12.2
BMI (kg.m^2^)	25.2±2.6	25.9±2.2	23.9±2.8
VO_2max_ (ml.kg.min^−1^)	50.7±5.9 (*n* =15)	52.0±5.1 (*n* =11)	47.1±7.2 (*n* =4)
Years of ultra running	2.7±1.2	2.8±1.4	2.6±1.0
Ultras completed^**^	9 (3–27)	8 (3–15)	11 (4–27)
24h races completed^**^	1 (0–3)	0 (0–3)	1 (0–1)
PRE-RACE DIET per 24h over 2days pre-race (*n*=16)^*^			
Energy (Kcal)	2,730±721	2,897±665	2,361±774
CHO (g)	296±87	309±86	267±90
CHO (g.kg^−1^)	4.0±1.4	3.8±1.2	4.4±1.8
Total fluid [food and drinks (ml)]	2,923±934	3,034±954	2,923±934
% Energy CHO	42±9	42±11	43±2
% Energy PROTEIN	17±4	17±4	16±3.0
% Energy FAT	36±9	35±10	39±5
% Energy Alcohol	9±11	11±14	5±5
PRE-RACE MEAL (1–4h pre-race; *n*=16)			
Energy (kcal)	878±349	858±317	921±447
CHO (g)	110±39	113±25	105±65
CHO (g.kg^−1^)	1.5±0.7	1.4±0.4	1.8±1.2
Protein (g)	29±14	28±16	33±9
Fat (g)	35±23	32±25	41±20
IN-RACE DATA			
Total race distance (miles)	80.6±15.7	84.0±13.5	75.3±18.5
Pace (mph)	3.8±0.5	3.8±0.4	3.7±0.6
Weight loss over race (%)	2.8±2.6	3.0±3.2	2.7±1.7
Mean HR (bpm; *n*=7)	124±11	-	-
HRmax (%)	68±5	-	-
VO_2max_ (%)	45±17	-	-

* Pre-race diet data includes pre-race meal. Values are mean (SD) except for

** which is reported as mean (range).

### Study Design and Data Collection

The study used an observational design, examining habitual dietary intake of participants before and during a 24-h race. No dietary intervention/advice was given prior to the study. Participants were asked to follow their usual pre-race and in-race diet routines. Pre-planned in-race feeding strategy was recorded on a questionnaire sent to participants ahead of the race day. Pre-race dietary intake was recorded using a weighed food intake method. Each participant was provided with electronic scales (Salter 1036, Tonbridge, United Kingdom) to weigh and record all foods/fluids consumed and also instructed to record all timings of intake. For anything consumed away from home, participants were asked to provide a description and estimate of portion size or send photographs to the researcher. In-race dietary intake was monitored by recorders assigned to each participant. Each participant had a base in the start-finish area, and digital scales (Salter 1036) were used to weigh foods/fluids consumed, recorded to the nearest 1g. Everything consumed at the start-finish area and food/fluid consumed during each lap was recorded along with each lap time. Water was available at the halfway point each lap; any water taken was self-reported and recorded.

Participants’ interstitial glucose concentration was monitored throughout the race using indwelling continuous glucose monitors (CGM; Abbott Freestyle-Libre), inserted into the subcutaneous tissue layer of the upper arm either on the evening before, or at least 2h before the event start on the morning of G24. CGMs automatically recorded interstitial fluid glucose concentration every 15min throughout the event. Manual readings of interstitial glucose were also obtained from participants each lap using a hand-held scanning device linked to the CGM. This device has been validated against blood glucose readings as reported in an FDA report (FDA, 2016).

Participants body mass was obtained post-void, in minimal under clothing, 1h prior to starting and after finishing (before any further food or fluid was consumed) to assess mass loss. A sub-set of participants (*n*=7) wore a heart rate monitor (HRM) and/or GPS device to track intensity in relation to HRmax and VO_2max_. All participants wore a timing chip which recorded lap times and total distance covered.

### Nutritional Analysis

All dietary intakes were analysed by a Registered Dietitian (EK), using Nutritics dietary analysis software (Nutritics Limited, Dublin, Ireland). Any food/fluids not available in the database were identified from manufacturer’s labels/information and added to the database. Dietary intake was analysed from 48-h pre-race weighed intake sheets to provide two sets of dietary data: (1) mean 24-h intake (pre-race diet); and (2) intake for 1–4h prior to race start (pre-race meal). A third set of dietary intake data came from analysis of each participant’s in-race diet. All macronutrients and energy intake were calculated (total, per hour, and per kg), total fluid (from foods and fluids), sodium, and caffeine. Individual intake of sugars (glucose, galactose, sucrose, fructose, maltose, and lactose) and starch or maltodextrin were also examined to investigate MTC intake. Fibre and oligosaccharide components were excluded from the individual carbohydrate analysis. Dietary intake was extrapolated from data per lap to represent nutrient intake per hour.

To investigate participants’ MTC intake, individual carbohydrate components were grouped according to those absorbed *via* the Sodium-Glucose co-transporter 1 [SGLT1; glucose, galactose, 0.5 x sucrose (glucose component), maltose, lactose+starch (to include maltodextrin)], and Glucose Transporter 5 [GLUT5; fructose+0.5×sucrose (fructose component)] transporters.

### Statistical Analysis

The main dependent variable was total distance covered, and this was regressed to independent variables including dietary intakes and other factors (VO_2max_, mass, BMI, and gender). Pearsons correlation coefficients, linear regression analysis, and independent *t*-tests were used to establish any associations between pre-race diet, pre-race meal, in-race diet, fitness, and ultra-running experience and distance achieved. Preliminary analyses were performed to ensure there was no violation of the assumption of normality, linearity, and multicollinearity. For independent *t*-tests, the sample was grouped according to G24 pre-race diet CHO·kg^−1^: those who consumed ≥5g·kg^−1^ per 24-h (*n*=3) and those who consumed <5g·kg^−1^ (*n*=13). Five grams per kg was selected as the lower-level recommendation for moderate exercise (Burke et al., 2011). Another divide was made with G24 in-race CHO intake, grouping the sample into those who consumed ≥40g·h^−1^ (*n*=6) and <40g·h^−1^ (*n*=12), in line with previous hourly in-race intake of amateur ultra-runners (Stellingwerff, 2016). Statistical significance was set at *p*<0.05, and effect sizes were measured using Hedges *g* with values of 0.2 considered a small effect, ~0.5 considered a medium effect, and>0.8 a large effect. For correlation coefficients, >0.5 and >0.7 were used to represent moderate and strong associations, respectively. A standard multiple regression analysis was performed to assess the ability of pre-race CHO and in-race CHO intake to predict race distance. Data are reported as mean (SD). Data were analysed using SPSS (IBM SPSS Statistics for Windows, Version 23).

## Results

The average temperature for G24 was 16±4°C (19°C maximum, 10°C minimum) with zero precipitation.

### Race Distance and Intensity

**Figure 1:**
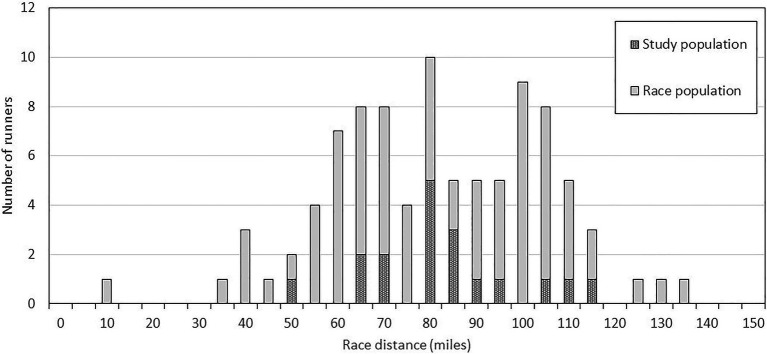
Histogram showing the spread of study participants across the G24 race population highlighting a representative sample of participants.

The leading male and female of the G24 study group covered 110.3 (177.5km) and 108.2miles (174.1km), respectively. The mean (range) distance covered by participants was 80.6±15.7 (48.0–110.0) miles [129.7±25.3 (77.2–177.5) km]. Ten of the participants continued moving for the full 24-h, six stopped to sleep (for between 3 and 8h), and two were unable to continue (one female stopped after 12h due to injury, one male after 19h due to gastrointestinal issues). The spread of study participants (*n*=18) within the race population was representative of participants across the field of competitors (total entrants *n*=89; Figure 1).

The sub-set of participants (*n*=7) who wore HRMs/GPS devices throughout the event exercised at a mean intensity of 68±5% HR_max_, equating to approximately 45±17% VO_2max_. (Table 1), thus representing low to moderate intensity exercise. Participants’ (*n*=15) VO_2max_ was positively associated with distance covered (*r*=0.58; *p*=0.02). To investigate VO_2max_ and distance further a linear regression was calculated, and a significant regression equation was observed [*F* (1,13) =6.73, *p*=0.02] with an *R^2^* of 0.341.

### Pre-race Diet

Sixteen of the *n*=18 participants completed adequate pre-race dietary monitoring. Participants’ mean consumption of CHO·kg^−1^ over the 2days pre-event was 4.0±1.4g·kg^−1^ per 24-h, contributing 42±9% to total energy (Table 1). A strong, positive association was identified between race distance and mean total CHO ingested in the pre-race diet (*r*=0.78; *p*<0.01) and also for CHO·kg^−1^ in the pre-race diet (*r*=0.70; *p*<0.01). Moderate positive associations were also observed between distance and mean energy intake (*r*=0.57; *p*<0.05) and energy·kg^−1^ (*r*=0.56; *p*<0.05). No associations were found between pre-race fluid intake and distance. An independent samples *t*-test identified a significant difference in distance covered [98.5±18.7miles (158.5±30.1km)] vs. [78.0±13.5miles (125.5±21.7km)] by participants who consumed ≥5g·kg^−1^ CHO per 24h in their 2-day pre-race diet vs. those who consumed <5g·kg^−1^; *t* (14)=2.23, *p*=0.042, Hedges’ *g*=1.43. This was also true for CHO·kg^−1^ 1-day pre-race [92.8±15.9miles (149.3±25.6km) vs. 75.2±12.9miles (121.0±20.8km)], respectively; *t* (14)=2.42, *p*=0.03, Hedges’ *g*=1.32.

### Pre-race Meal

All participants consumed food and fluid in the 1–4h pre-race with a mean energy intake of 878±349kcal and CHO intake of 1.5±0.7g.kg^−1^ contributing 49±15% of total energy intake. Mean fluid intake was 940±397ml. A moderate positive association was observed between race distance and total CHO in the pre-race meal, *r*=0.68, *p*<0.01 and also with CHO.kg^−1^, *r*=0.57 *p*<0.05.

### In-Race Diet

#### Energy Intake

Total energy consumed, by participants was 3,907±1,658kcal with a mean of 179±63kcal·h^−1^. Energy intake composed of 69% CHO (721±326g), 8% protein (78±49g), and 21% fat (90±55g). A wide variety of foods, fluids, and commercially available sports-nutrition products were consumed in-race (Table 2). Fifteen participants (83%) consumed sports-nutrition products (gels/bars/sports-drinks). In this sub-section, sports-nutrition products contributed 22±14% of total energy and 28±15% of total CHO intake. There were no differences in energy, macronutrients, sodium, or caffeine intake between genders. A moderate positive association was observed between distance achieved and total energy intake during the event when corrected for body mass (*r*=0.52; *p*=0.028).

**Table 2:** Nutritional composition (mean±SD) of all foods and fluids consumed by participants over the 24-h race duration (in-race diet), and actual foods, fluids, and sports-nutrition products consumed by *n*=18 participants during the Glenmore 24 trail race.

Variable	All Participants (*n*=18)	Males (*n*=11)	Females (*n*=7)
Total Energy (Kcal)	3,907±1,658	4,407±1,432	3,123±1,788
Energy (Kcal.h^−1^)	179±63	201±55	144±63
Energy (Kcal.kg^−1^)	52±23	54±17	50±33
Energy-1st 12h (Kcal.h^−1^)	207±86	236±73	162±90
Energy-2nd 12h (Kcal.h^−1^)	148±54	156±58	133±49
Total CHO (g)	721±326	828±288	551±328
CHO (g.h^−1^)	33±12	38±11^*^	26±12
CHO – 1st 12h (g.h^−1^)	39±18	45±16	30±18
CHO – 2nd 12h (g.h^−1^)	26±9	28±8	22±8
CHO (g.kg^−1^)	9.6±4.5	10.1±3.3	8.8±6.1
Total Protein (g)	78±49	80±46	73±57
Protein (g.h^−1^)	3.6±1.9	3.6±1.8	3.5±2.2
Total Fat (g)	90±55	98±55	78±57
Fat (g.h^−1^)	4.1±2.3	4.5±2.4	3.6±2.2
Total Fluid [food and drinks (ml)]	6,920±2,004	8,047±1,461^*^	5,149±1,352
Fluid (ml.h^−1^)	326±92	371±73^*^	255±76
Total sodium (mg)	4,217±2,241	4,589±1,907	3,633±2,741
Sodium (mg.h^−1^)	195±95	212±88	169±105
Sodium (mg.kg^−1^)	56.3±32.7	56.3±22.9	56.2±46.4
Total Caffeine (mg)	247±141	287±133	184±139
Caffeine (mg.h^−1^)	11.4±6.7	13.4±6.8	8.2±5.7
Caffeine (mg.kg^−1^)	3.2±1.6	3.5±1.5	2.7±1.7
FOODS, FLUIDS, and SPORTS-NUTRITION PRODUCTS CONSUMED BY G24 PARTICIPANTS IN-RACE			
Foods (savoury) consumed	Mixed nuts, bagels, quiche, corned beef hash, soup, porridge pots, pasta pots and sachets, pot noodles, Weetabix and milk, fish and chips, pork pies, ryvita, avocado, stew, cheese, ham, bread and butter, crisps, croissant, pizza, smoked sausage, fried eggs, butteries, rice cakes, and peanut butter.		
Foods (sweet) consumed	Fruit–Dried, fresh, tinned in juice, fruit and jelly pots, flapjack, rice pudding, custard pots, sweets (boiled, chewy, jelly, and fudge), mints, chocolate bars, iced buns, Eat Natural bars, iced buns, dextrose tablets, cereal bars and biscuits (chocolate and plain), cereal, yoghurt, baby food sachets, muffins, and malt loaf.		
Fluids (non-sports)	Beetroot juice, Coconut water, water, tea, coffee, cola, Irn Bru, milkshake, hot chocolate, Dioralyte, ginger ale, Sprite, Innocent smoothies, Red Bull, milkshake, Sugar free diluting juice, and homemade energy drink (13% CHO solution: maltodextrin/glucose/fructose).		
Sports nutrition products	Gels, sports beans, Shot Bloks, Tailwind, Lucozade Sport, Gatorade, SIS isotonic, Clif bars, Chia Charge bars, Power bar Energise, Powerade, Nuun electrolyte, High 5 zero, Protein shakes, Mountain Fuel Extreme, and S!Caps.		

Data are mean±SD.

* Mean value was significantly different to female runners (*p*<0.05).

#### Total Carbohydrate Intake and Interstitial Glucose Profiles

**Figure 2:**
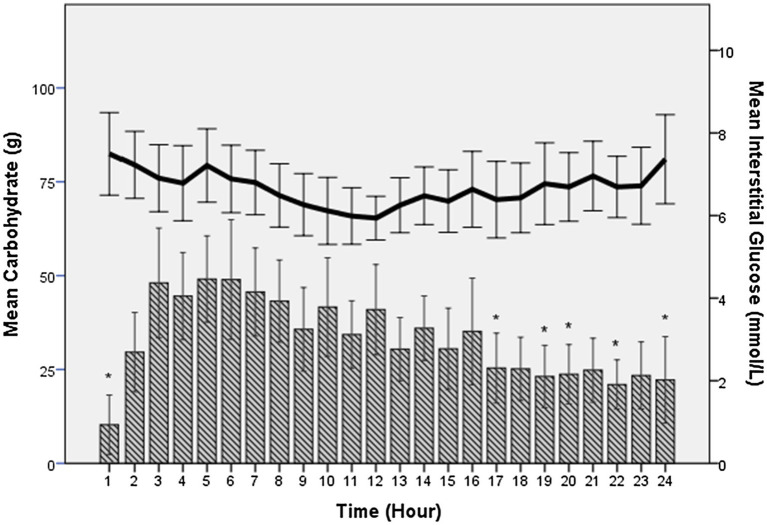
Hourly mean interstitial glucose concentration (continuous line/right axis) and hourly mean carbohydrate (CHO) intake (bars/left axis) for participants during the G24 ultra-endurance race. Mean hourly CHO intake peaked at 49±6g in hour 5. ^*^indicates significantly different hourly intakes to peak value. No significant differences were observed over time for mean interstitial glucose concentration.

Mean hourly intake of CHO for all participants was 33±12g·h^−1^. During the event, CHO intake peaked in hour 5 at 49±6g with significantly lower amounts consumed in hours 1, 17, 19, 20, 22, and 24 (Figure 2). Individual hourly consumption varied widely with 67% of participants (*n*=12) taking between 60 and 90 g·h^−1^ on at least one occasion, and 17% (*n*=3) taking in excess of 100g·h^−1^ at least once. There was a significant difference in hourly CHO intake [38 vs. 26g; *t* (16)=2.27, *p*=0.037] between males and females, respectively, but not when corrected for body mass (0.5±0.1 vs. 0.4±0.2g·kg^−1^·h^−1^).

Fourteen participants retained CGM sensors for the entire race duration. No association was observed between mean interstitial glucose concentration and dietary CHO intake, or with race distance. Glucose profiles were variable throughout the event. Average hourly glucose concentration for all participants ranged from 3.1 to 13.4mmol·l^−1^, indicating times of both hypo and hyperglycaemia during the race. Overall mean glucose for participants who retained CGM devices for the full race duration was 6.9±1.2mmol·l^−1^ (Figure 2). Unpublished data from our own laboratory indicate that interstitial glucose readings are elevated above blood glucose concentration during moderate intensity exercise by ~2mmol·l^−1^ (Wilson and Galloway, unpublished observations) and readings can be influenced by exercise (FDA, 2016).

#### Multiple Transportable Carbohydrate Intake

Participants consumed CHO at a mean rate of 0.6±0.2g·min^−1^, less than the 1g·min^−1^ needed to saturate SGLT1 transporters (Jeukendrup, 2010). Estimated intake of individual CHO components was as follows: starch (12.7±7.1g·h^−1^); sucrose (6.2±4.3g·h^−1^); glucose (3.5±1.5g·h^−1^); fructose (3.6±2.3g·h^−1^); galactose (0.01±0.03g·h^−1^); maltose (0.4±0.4g·h^−1^); and lactose (0.9±0.9g·h^−1^) with fibre and oligosaccharide likely making up the remaining amount. Mean intake ratio of glucose: fructose equivalents were 3±2:1, (range 2:1–8:1). Estimated mean sugars available for absorption per hour *via* SGLT1 and GLUT5 transporters were 21±9g, (range 8–39g) and 7±3g, (range 2–15g), respectively. Hourly transport capacity was not reached for either carbohydrate transporter with a notional remaining capacity for participants of around 39g·h^−1^ for SGLT1 and around 22g·h^−1^ for GLUT5.

#### Carbohydrate Dose and Distance

**Figure 3:**
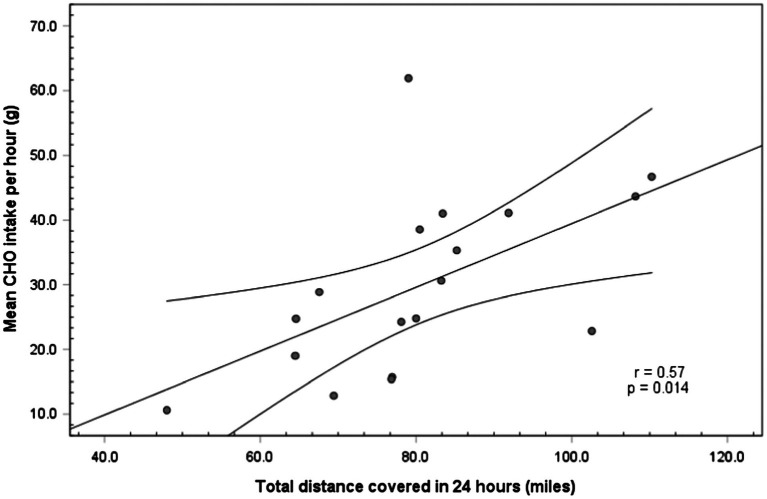
Total race distance covered vs. CHO intake (g·h^-1^) during 24h races for G24 runners showing a moderate positive association.

Moderate positive correlations were observed between distance and in-race total CHO, *r*=0.65 and total CHO·kg^−1^, *r*=0.64 (both *p*<0.01). A significant difference in distance was observed between those consuming ≥40g·h^−1^ [92.2±13.9miles (148.4±22.4km)] and <40g·h^−1^ [74.7±13.5miles (120.2±21.7km)]; *t* (16)=2.56, *p*=0.021, Hedges’ *g*=1.28. A moderate positive relationship between in-race CHO (g·h^−1^) and distance was observed (*r*=0.57, *p*=0.01; Figure 3).

A significant regression equation was found [*F* (2,13)=12.2, *p*=0.001], with an *R^2^* of 0.653. In this sample of participants, both pre-race diet and in-race CHO variables were significant predictors of race distance. Participants’ race distance increased by 6.6miles (10.6km), 95% CI, 2.16–11.1miles (3.5–17.9km) for each 1g·kg^−1^ CHO in pre-race diet and 1.5miles (2.4km), 95% CI, 0.19–2.86miles (0.3–4.6km) for each 1g·kg^−1^ CHO in-race when other variables remain constant. Although significant correlations with distance were found between V0₂max, pre-race energy intake, pre-race meal CHO, and in-race energy·kg^−1^, these variables were not significant predictors of distance in the final regression model.

#### Ultra-Experience and Diet/Distance

For the pre-race diet, moderate positive correlations were observed between years of ultra-running and mean CHO intake, *r*=0.57 and mean CHO.kg^−1^, *r*=0.53 (both *p*<0.05). Number of ultras completed was also positively associated with pre-race diet CHO/kg, *r*=0.62 (*p*<0.05). For the in-race diet, no significant associations were observed between ultra-experience and CHO consumption but interestingly there was a moderate negative association between number of ultras completed and energy intake per hour *r*=−0.50 (*p*<0.05). No association was found between ultra-running experience and distance achieved.

#### Fluid Intake/Dehydration:

Fluid intake (foods and fluids) per hour [371 vs. 255ml; *t*(16)=3.25, *p*=0.005] and total fluid intake [8,047 vs. 5,149ml; *t*(16)=4.22, *p*=0.001] were significantly different between males and females (Table 2), but this was not the case when corrected for body mass (4.6±1.1 vs. 3.9±1.2ml·kg^−1^·h^−1^/100±24 vs. 79±26ml·kg^−1^). Body mass loss over the race was 2.8±2.6% and no association was found between fluid intake and race distance covered.

## Discussion

The aims of this study were to observe the diet of ultra-endurance runners prior to and during a field-based 24-h trail race, to compare observations with current recommendations for CHO intake and to determine intake of MTC’s. The main findings were that CHO intake was lower than current recommendations for pre-race diet and in-race intake. CHO intake was significantly related to distance achieved in the event and, based on CHO sources ingested, runners had capacity to increase their intake of MTC to help them achieve recommended CHO intakes. For both pre-race diet and in-race intake, those who consumed more CHO per kg body mass achieved greater overall race distances.

### Pre-race Diet and Pre-race Meal

The pre-race diet observations demonstrate that athletes were only meeting the fuelling recommendations for short duration low intensity activities. Thus, promoting a higher CHO intake over 48-h pre-race could significantly influence race performance, although a direct cause and effect relationship cannot be confirmed from the present study due to the lack of specified intervention and control groups. Additional CHO in the region of 1–2g/kg per 24-h in the pre-race diet represents an initial realistic and achievable adjustment to CHO intake for these ultra-runners. However, an increase of this magnitude would only take these athletes into the 5–7g/kg per 24-h range, just more than half of the recommended CHO intake for fuelling very prolonged moderate intensity exercise events. In the present study, although ultra-running experience was associated with a higher CHO intake, it is not known if pre-race CHO intake was higher than habitual CHO intake, or if participants actively carbohydrate-loaded, but their intake was below current recommendations. These pre-race CHO intake observations are similar to those of competitors before an 85-mile mountain-marathon who consumed 4.5±1.1g·kg^-1.^day^−1^ pre-event (Mahon et al., 2014), where 86% of participants planned to increase CHO over these final days. Atkinson et al. (2011) reported an intake of 5.0±1.9g.kg^−1^ CHO the day before a marathon with 68% of participants claiming to have adopted “high-carbohydrate diets.” Atkinson et al. (2011) also observed that CHO content in the pre-race diet was an important predictor of marathon finishing time. Collectively, these observations could indicate that individuals do not know how to carbohydrate-load effectively, or that they have a low habitual intake of CHO. The current study supports the need for education on CHO loading strategies to help ultra-distance runners achieve more beneficial CHO intakes in their pre-race diet (Costa et al., 2014; Beck et al., 2015). For the pre-race meal, the athletes managed to meet current CHO intake guideline (Thomas et al., 2016) and intake during this 4-h pre-event period showed a significant association with race distance. However, an increased intake of CHO could still be achieved within the 1–4g.kg^−1^ recommendation. Therefore, it seems that a greater emphasis and education placed on meeting CHO intake guidelines within the pre-race diet/meal would be beneficial to their performance in ultra-running events.

### In-Race Intake

In the present study, mean in-race CHO intake was in the 30–60g·h^−1^ range for the runners, but fell short of guidance for up to 90g·h^−1^. Hourly in-race CHO intake was low compared to other studies on prolonged ultra-endurance running (12h plus) including both amateur finishers (66g·h^−1^), non-finishers (42g·h^−1^; Stuempfle et al., 2011), elite-runners (71g·h^−1^; Stellingwerff, 2016) in 100-mile mountain races, runners in a 100-mile trail race (54g·h^−1^; Glace et al., 2002), and a 24-h track world championship (62g·h^−1^; Lavoué et al., 2020). However, Costa et al. (2014) recorded similar CHO consumption rates (37±24g·h^−1^) to the present study for participants during the same G24 event in 2011/2012. Likewise, Martinez et al. (2018) recorded CHO intakes of 32±15g·h^−1^ in ultra-endurance mountain runners over three distances (27/41/70-miles). Lower intake (28±17g·h^−1^) also was observed in the study by Mahon et al. (2014), which may have been due to runners carrying all food and fluids and wanting to minimise additional weight. CHO intake therefore appears to rarely reach 90g·h^−1^ in these types of ultra-endurance running events.

Mean in-race CHO intake at 33±12g·h^−1^ in the present study would not be sufficient to saturate SGLT1 transporters and therefore intake rate would not be limiting to CHO absorption. In race CHO intake could be elevated through intake of a variety of CHO sources to push runners towards the 90g·h^−1^ recommendation, with an increase of 10–20g·h^−1^ from isotonic fluids, cereal bars, sports gels, incorporating maltodextrin or glucose, and fructose probably being achievable by most runners. As research knowledge builds on MTC use and more evidence emerges on ideal ratios of carbohydrate types or delivery methods for improved gut tolerance, ultra-runners would benefit from education around increasing CHO from a variety of sources into their race strategies.

Stellingwerff (2016) highlights consistent variation in CHO consumed in-race by elite (61g·h^−1^) and amateur ultra-runners (41g·h^−1^) when collectively comparing previous studies, highlighting that 20g·h^−1^ difference can lead to substantial deficits in CHO and energy over events. Relating this to the present study, an additional 20g·h^−1^ would amount to a difference of 480g CHO and 1,800 kcals over 24-h. An intervention study on marathon runners demonstrated the effect of this CHO gap. Runners were grouped into those with an intervention target of 60g·h^−1^ maltodextrin/glucose (actual intake 64.7±12.3g·h^−1^) and those who chose CHO freely (actual intake 38.0±17.5g·h^−1^). The intervention group demonstrated 5% faster finishing times (Hansen et al., 2014), suggesting that the extra CHO resulted in improved performance. Future intervention studies could investigate the performance effect of bridging this CHO gap in amateur ultra-runners.

Hourly energy and CHO intake fluctuated throughout the event, with lower intakes towards the end. The impact of fatigue on motivation to eat and drink was clear in the G24 runners. Experienced support crew is invaluable in helping runners to meet nutritional targets and cajole when psychologically low. This support crew can make the difference between achieving a successful outcome or not (Holt et al., 2014). Normal circadian variation also could be a factor in the decline in oral intake observed overnight (Serin and Acar Tek, 2019). Between 2 and 6am, a circadian low is experienced, which results in a difficult time for ultra-endurance competitors. In G24, more participants (28%, *n*=5) stopped to sleep during these hours than at other times. An interesting question would be to explore whether runners could train themselves to eat more during these hours, and whether additional food intake could influence their decisions to continue or rest, and ultimately impact upon distance achieved.

Analysis exploring the role of in-race CHO on race outcome demonstrated that those consuming ≥40g·h^−1^ ran further than those consuming <40g·h^−1^. However, it is unlikely that these differences were due to CHO intake alone, as other factors such as VO_2max_ and years of ultra-marathon experience also are likely to impact on race outcome. Indeed, a moderate positive association between distance and VO_2max_ was observed in the present study suggesting that cardiovascular fitness is likely to be a confounder, with fitter runners running faster/further. Fitter/faster athletes also would have higher CHO requirements and/or be more aware of nutritional recommendations, meaning they would likely consume more CHO than slower athletes (Havemann and Goedecke, 2008).

### Activity Intensity/Interstitial Glucose Concentration

A sustained intensity of 45±17% VO_2max_ demonstrates that, in ultra-events, exercise intensity is low to moderate, but when sustained over 24h this becomes a significant metabolic challenge. Other studies have reported low mean heart rates in ultra-endurance running events (Clemente-Suarez, 2015; Stellingwerff, 2016) and low pace (Glace et al., 2002; Clemente-Suarez, 2015; Ramos-Campo et al., 2016). It therefore could be suggested that in-race CHO recommendations for ultra-runners need not be high, given that endogenous fat stores will likely contribute significantly to energy requirements, and total CHO oxidation rates will be lower at lower intensities (Jeukendrup, 2014). However, an adequate amount of exogenous CHO is important to conserve muscle and liver glycogen and maintain blood glucose particularly under the challenging demands of a 24-h event. To achieve this without GI distress likely requires a good balance of MTC intake alongside other macronutrients to support total energy requirements.

Although no associations were observed between interstitial glucose levels and dietary intake, it was curious to see the variations in participants’ glucose profiles. Mean glucose concentration was 7.2mmol·l^−1^ initially, with a nadir of 5.9mmol·l^−1^ mid-race, rising to 7.4mmol·l^−1^ after 24h. From Figure 2 there appears to be an inverse relationship, with interstitial glucose concentration declining as CHO intake is higher over the first 12h, and rising latterly as CHO intake declines. This could be a response to circadian hormonal control of glucose concentration. Ramos-Campo et al. (2016) tested runners’ blood glucose pre (5.1±0.5mmol·l^−1^) and post (5.8±1.4mmol·l^−1^) a 54km mountain race, showing little variation but no indication of how glucose levels responded during the race. Similar, steadier blood glucose concentrations than in the current study were observed in runners before (5.0), during (5.4), and after (5.3mmol·l^−1^) a 100mile trail race (Glace et al., 2002). To the researchers’ knowledge, this is the first study to monitor interstitial glucose during a competitive ultra-endurance running event, with glucose readings reflecting the lag time between blood and interstitial glucose. Future studies using CGM devices should investigate corresponding changes in hormone concentrations such as insulin and cortisol, or monitor the effect of specific rates of CHO ingestion on glucose concentration to decipher the primary determinants of fluctuations.

### Future Considerations

Whilst no firm guidance can be established from this study, the findings do support the importance of both pre-race and in-race CHO intake on performance in a 24-h race. Future research should test these experimentally under field-conditions using increased intake of MTC’s, perhaps making use of newer products containing alginate hydrogel to deliver higher rates of MTC with minimal GI distress (Sutehall et al., 2018) when ‘food fatigue’ occurs in later stages of a 24-h race. In addition, investigating feeding strategies in ultra-endurance runners matched for VO_2max_, would help to establish if increasing quantities of pre-race and in-race CHO result in performance improvements. It should be noted that intake of MTCs was difficult to calculate accurately in the present study due to the restricted proprietary nutritional information of specific sugar configurations in some sports-nutrition products. However, the current observations do support recommendations to increase CHO intake in preparation for, and during, ultra-endurance events, and provide insight into the range of carbohydrate sources that could be ingested to help meet target intakes of MTC’s.

## Conclusion

In this study, the amount of ingested CHO both during the pre-race diet and in-race was lower than current recommendations. Given the duration of the event, despite a low to moderate intensity of exercise, total energy requirements are very high. Therefore, ultra-endurance athletes need to consider ways to increase energy and CHO intake prior to and during these types of events. Our analysis suggests that this can most easily be achieved through increasing pre-race diet carbohydrate intake, and working on strategies to enhance intake of MTC’s up to 90g/h in-race. Strategies could include improved education on carbohydrate loading in the days prior to an ultra-endurance event and/or the incorporation of additional sports nutrition products composed of maltodextrin/fructose in-race. Making use of novel products containing alginate hydrogels, especially in the later stages of a 24-h event when dietary intake is most difficult could prove beneficial.

## Data Availability Statement

The raw data supporting the conclusions of this article will be made available by the authors, without undue reservation.

## Ethics Statement

The studies involving human participants were reviewed and approved by University of Stirling ethics committee. The patients/participants provided their written informed consent to participate in this study.

## Author Contributions

EK and SG conceived the study, undertook data collection and analysis, and contributed to writing the manuscript. All authors contributed to the article and approved the submitted version.

## Conflict of Interest

The authors declare that the research was conducted in the absence of any commercial or financial relationships that could be construed as a potential conflict of interest.

## Publisher’s Note

All claims expressed in this article are solely those of the authors and do not necessarily represent those of their affiliated organizations, or those of the publisher, the editors and the reviewers. Any product that may be evaluated in this article, or claim that may be made by its manufacturer, is not guaranteed or endorsed by the publisher.
